# Management of rarely seen internal tunnelling root resorption associated with a maxillary permanent incisor

**DOI:** 10.1038/s41415-024-7504-7

**Published:** 2024-06-28

**Authors:** Kirsty A. Carney, Thibault N. E. Colloc, Julie K. Kilgariff

**Affiliations:** 4141594993001 Post Dental Core Training Fellow, Dundee Dental Hospital and Research School, Dundee, Scotland, UK; 4141594993002 Clinical Lecturer and Honorary Specialty Registrar in Endodontics, Dundee Dental Hospital and Research School, Dundee, Scotland, UK; 4141594993003 Consultant in Endodontics, Dundee Dental Hospital and Research School, Dundee, Scotland, UK

## Abstract

This case presents clinical techniques and materials that may be applied in the management of internal tunnelling root resorption, a rare condition which has little published literature.

A 25-year-old woman was referred to a specialist centre by her general dental practitioner following an incidental finding of a radiographic radiolucency, suggestive of root resorption associated with tooth 21. A provisional diagnosis of external cervical resorption was made, and following cone beam computed tomography (CBCT), a definitive diagnosis of internal tunnelling root resorption given. Non-surgical endodontic treatment was completed using continuous wave compaction with a calcium-silicate based sealer. No further intervention has been required.

A summary of the histology, aetiology, prevalence, diagnosis, management and prognosis of internal tunnelling root resorption is provided. The benefits of CBCT for diagnosing and managing internal tunnelling resorption defects and using continuous wave compaction with a calcium-silicate based sealer, compared to cold obturation techniques, are discussed.

**Supplementary Information:**

Zusatzmaterial online: Zu diesem Beitrag sind unter 10.1038/s41415-024-7504-7 für autorisierte Leser zusätzliche Dateien abrufbar.

## Key points

- Summarises the history, aetiology, prevalence, diagnosis, management and prognosis of internal tunnelling root resorption.
- Discusses the benefits of cone beam computed tomography for diagnosing and managing internal tunnelling root resorption defects.
- Describes the clinical techniques and materials that may be applied in the management of internal tunnelling root resorption.

**Supplementary Information:**

Zusatzmaterial online: Zu diesem Beitrag sind unter 10.1038/s41415-024-7504-7 für autorisierte Leser zusätzliche Dateien abrufbar.

## Introduction

Internal root resorption (IRR) is described as the progressive destruction of intra-radicular dentine and dentinal tubules along the middle and apical thirds of the canal walls from clastic activity.^1^ It is a relatively rare pathology and its aetiology and pathogenesis not completely elucidated.^2^ Different subtypes of IRR may present clinically with similar features but require different management depending on their transient or progressive nature.

### Histology

Histologically, IRR can present with different manifestations based on the pattern and extent of resorption, such as internal inflammatory resorption, internal surface resorption, or internal replacement resorption, as described in online Supplementary Table 1.^3^^,^^4^

### Aetiology

**Fig. 1:**
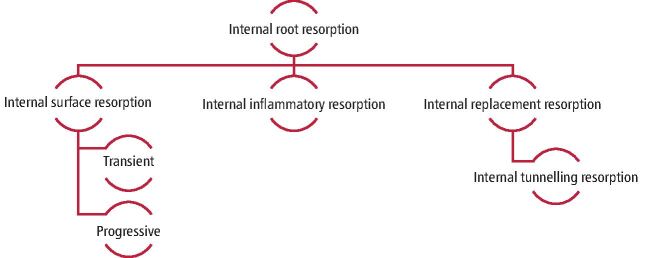
Subtypes of internal root resorption

Despite a lack of clear aetiology in the literature, an external stimulus inducing an inflammatory process in the pulp, such as a traumatic dental injury (TDI), or mechanical forces from orthodontic treatment, may lead to IRR developing.^5^ IRR is thought to be initiated by damage to the odontoblastic and unmineralised predentine layer, exposing mineralised radicular tissue to the pulp and resulting in the migration of odontoclasts from the pulp to the site of injury.^4^ The process requires two phases: a mechanical or chemical injury to the protective tissues and stimulation by infection or pressure. Similar injuries can lead to different types of internal root resorption as described in Fig. 1.

### Prevalence

The prevalence of IRR is not widely reported. Laboratory studies examining extracted teeth have found a higher incidence of IRR compared to studies using observation of radiographs, suggesting that IRR may be clinically and radiographically underdiagnosed. This may be attributed to the absence of clinical symptoms until later stages and to the size of resorption; small resorptive lesions are less likely to be detected by conventional radiographs.^6^

### Subtypes of internal root resorption

Different subtypes of IRR have been described in the literature (Fig. 1).^7^ Internal tunnelling resorption (ITR) is an uncommon variant of internal replacement resorption. Typical features include tunnelling resorption which burrows behind the predentine layer, adjacent to the root canal, with concommitant deposition of cancellous bone-like tissues. Since the osteoclastic activity is related to the healing activity, the resorption process can stop, and complete pulp canal obliteration may be seen on follow-up radiographs due to osteoblastic-like cell activity in the pulpal space.^8^ ITR is usually found in teeth with a history of root fractures but may also occur after luxation injuries if damage to the odontoblastic and unmineralised predentine layer occurs at the time of the TDI, inducing pulpal inflammation and odontoclast migration.^9^

### Clinical and radiographic findings

Symptomatic cases of progressive IRR may develop symptoms of pulpitis if the dental pulp is still partially vital. If the dental pulp becomes necrotic, signs and symptoms of symptomatic apical periodontitis or apical abscess, with possible presence of a sinus tract related to perforation of the resorption and/or a periapical lesion, may develop.^1^

Asymptomatic cases have been reported in the literature presenting as discolouration of the clinical crown eg a pink spot caused by granulation tissue shining through the dental hard tissues at the site of resorption in the coronal third of the root canal.^10^ However, it is more common for external cervical resorption (ECR) to present in this way.^11^Asymptomatic cases may present clinically with a sinus and a resorptive lesion identified following radiographic investigation.

**Fig. 2:**
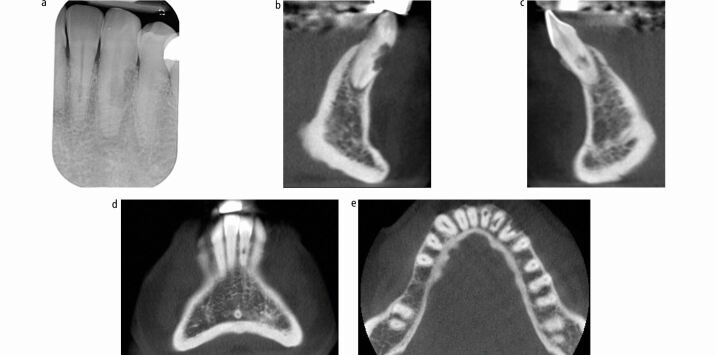
CBCT imaging in coronal, axial and sagittal planes provides information about the extent and location of IRR, overcoming 2D limitations, aiding formulation of an appropriate treatment approach. a) Pretreatment periapical of tooth 33 and 32 reveals an atypical radiolucency on the root. The labial and lingual extents of these lesions are unknown from this image. It is not possible to identify if the radiolucencies are from a defect within the root canal or on the external root surface. b) Pretreatment sagittal view of CBCT demonstrating resorptive lesion in the coronal half of the root 33. There is loss of much of the labial surface and the defect involves the canal. This tooth has a guarded prognosis. This information allowed an informed discussion with the patient as to treatment options, risks, benefits and prognosis. c) Pretreatment sagittal view of CBCT demonstrating internal root resorption of 31 with no obvious perforation. d) Pretreatment coronal view of CBCT demonstrating internal root resorption of 31 with no obvious perforation. e) Pretreatment axial CBCT view demonstrating root resorption 31, 32, 33, with resorption perforating 33 on the buccal surface and 32 on the mesial surface. Knowledge regarding the extent of the resorptive defects has informed the treatment approach. 31, least affected by resorption, will be treated with a non-surgical approach. 32 and 33 treatment will most likely involve a surgical and non-surgical approach

The diagnosis of root resorption can usually be confirmed using a parallax radiograph technique.^12^ ITR may be differentiated from other types of IRR by the pattern of hard tissue deposition. Two-dimensional images can have superimposition and limitations in diagnosing the nature, severity and location of the resorption. The extent of the resorption in all three planes and the spread within the roots may not be captured (Fig. 2).^13^ CBCT provides 3D information, aiding diagnosis and treatment planning when used in conjunction with clinical assessment. CBCT is reportedly more accurate for diagnosing and determining the extent and location of resorptive defects compared with periapical radiographs following dental trauma.^14^

A position statement from the European Society of Endodontics (ESE) recommends CBCT examination be considered for the assessment and/or management of root resorption, which clinically appears potentially amenable to treatment.^6^^,^^15^ High resolution CBCT in endodontics allows assessment of finer anatomical details. The volume of exposed tissue and effective radiation dose can be minimised by reducing the field of view, reducing scatter and improving image quality.

**Fig. 3:**
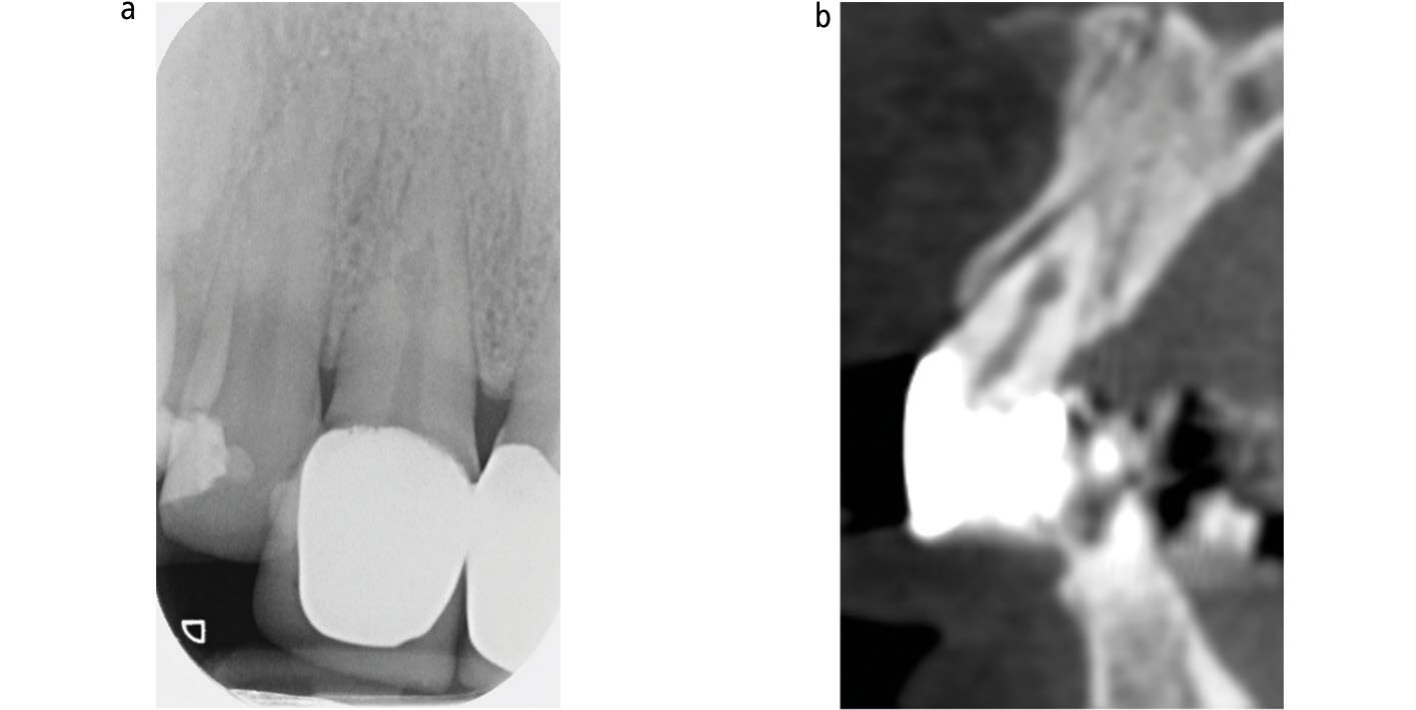
A case where periapical radiographs and CBCT were used to identify the tooth surface(s) affected and extent of the resorptive defect. a) The pretreatment periapical of tooth 11 reveals a radiolucency over the mid-third of the root canal system. The labial and palatal extent of this lesion is unknown from this image. It is not possible to identify if the radiolucency is from a defect within the root canal or on the external root surface, or indeed if there is a communication (perforation) from the extent of the resorption. b) The pretreatment CBCT image confirms the resorption is within the root canal system (IRR) and does not perforate. This information is used to formulate an appropriate treatment approach (non-surgical)

IRR and ECR can also have similar radiographic appearances. IRR is commonly described as an oval radiolucency centred on the root canal as can ECR at the cervical area. To identify the tooth surface(s) affected, the extent and to differentiate internal from external resorptive defects, parallax radiographic techniques and/or the use of cone beam computed tomography (CBCT) may be necessary (Fig. 3).^16^^,^^17^^,^^18^

### Management of internal root resorption

IRR, depending on its aetiology and pattern, can be described as transient and self-limiting or progressive. Transient IRR may occur immediately following an injury and spontaneously resolve and halt. Such lesions, once identified, should be monitored yearly, clinically and radiographically, to ensure no further progression occurs. Progressive IRR will continue to develop unless pulp necrosis or an intervention occurs. A recent ESE position statement describes the recommended management of progressive IRR, which includes ITR.^3^ The treatment options are: in the absence of resorption perforating, non-surgical endodontic treatment, or, when resorption has perforated, non-surgical endodontic treatment ± internal or external (surgical) repair of the perforation defect; alternatively, tooth extraction.

ITR can be managed through non-surgical endodontic treatment when the defect is contained within the root. CBCT may be required to confirm if there is perforation. Perforation complicates management because periodontal tissues become involved, inflamed, and challenges ensue in the preparation, disinfection and obturation of a root canal system in communication with periodontal tissues. Accordingly, perforation of a resorptive lesion is unfavourable, reducing the prognosis and a surgical approach may be needed in addition to intracanal therapy.^19^ For these reasons, referral of IRR cases to a specialist is encouraged.

When non-surgical endodontic treatment is perfomed, conventional instrumentation and irrigation with sodium hypochlorite and a chelating agent alone may be insufficient to achieve adequate disinfection of the entire root canal system as the resorptive defect may be challenging to access, especially in ITR. Despite conflicting evidence,^20^ the use of dynamic irrigation to enhance cleaning of the root canal system, for example by using a combination of manual dynamic activation with a master gutta percha cone,^21^ or the use of an interdental brush in a wide canal,^22^ can improve the penetration of sodium hypochlorite into complex resorption defects. Sonic or ultrasonic activation can also be used to enhance the removal of dentine debris and organic tissue.^23^ Following adequate instrumentation and disinfection, the use of calcium-silicate-based sealers (CSBS) is beneficial for several reasons in comparison to resin-based sealers. Epoxy resin-based sealers have become considered the gold standard due to dimensional stability and resorption resistance.^24^ A limitation of resin-based sealers is incomplete filling of the root canal due to the hydrophobicity, whereby moisture in the root canal and/or dentinal tubules can result in suboptimal adhesion to the root canal system walls.^25^ Superior flow properties of CSBSs are beneficial as haphazard deposition of cancellous bone-like tissue in ITR can result in irregularities in the root canal space. Furthermore, CSBSs are very biocompatible/bioactive and can stimulate hard tissue formation and reformation of cellular cementum at the anatomical apex.^26^ CSBSs are less likely to induce subsequent inflammation in the periapical region.^27^

This case illustrates the challenges in diagnosing and managing ITR and the treatment of an ITR case using modern equipment, materials and techniques.

## Case report

**Fig. 4:**
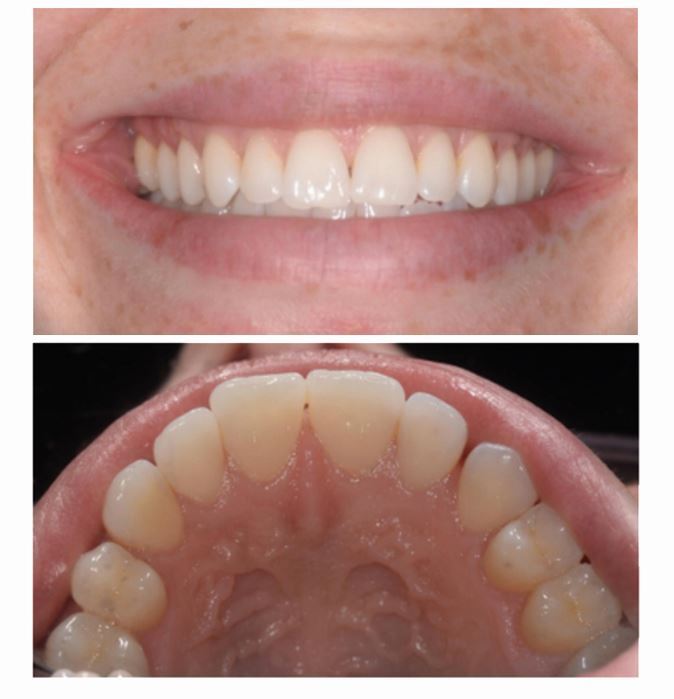
Pretreatment clinical photographs

**Fig. 5:**
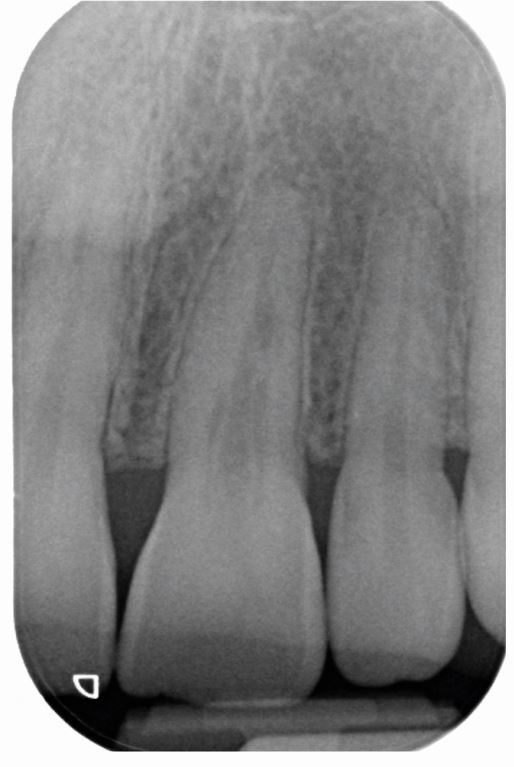
Preoperative periapical radiograph of tooth 21, taken 11 April 2022, showing root end blunting and the appearance of resorption in the mid-third of the root

**Fig. 6:**
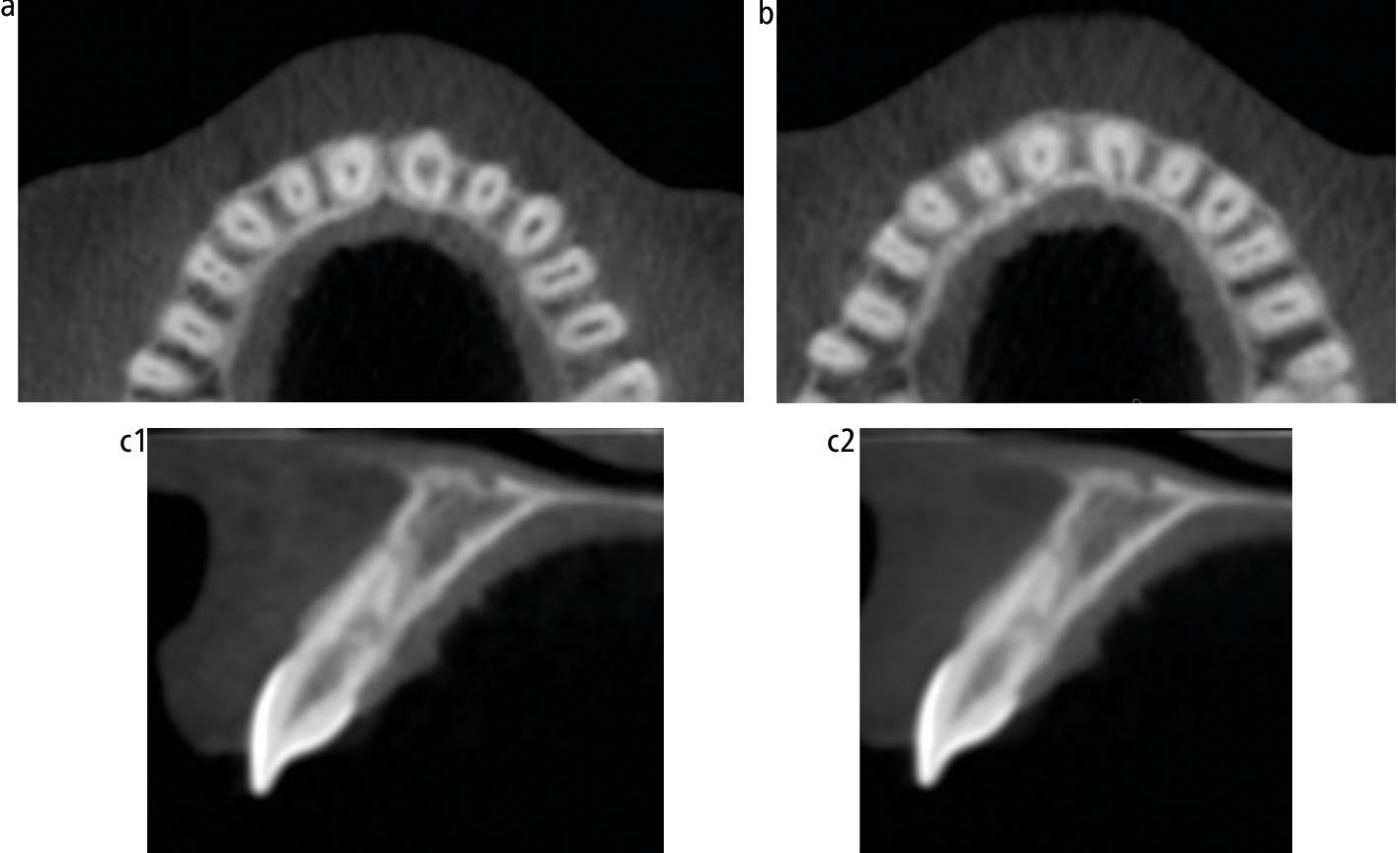
a) Pretreatment axial view of CBCT showing a resorptive defect on tooth 21, with enlargement of the root canal system in the mid-third of the root. b) Pretreatment axial view of CBCT showing a resorptive defect on tooth 21, with radiolucent channel and clear exposure of the root canal system at the mid-third of the root canal on the palatal aspect of the tooth. c) Pretreatment sagittal view of CBCT showing palatal extent of resorptive defect on tooth 21 with a disorganised pattern

A 25-year-old woman was referred by her general dental practitioner in March 2021 to a teaching hospital and dental specialist centre, regarding an incidental finding of root resorption associated with tooth 21. The patient was fit and well, a lifelong non-smoker and consumed alcohol within recommended limits. The dental history was unremarkable: tooth 21 was asymptomatic, with no history of dental trauma. Fixed orthodontic appliance therapy was completed eight years previously and a periapical radiograph taken to plan clear aligner therapy revealed an irregular radiolucency associated with the root of tooth 21. The patient had an unrestored dentition with Class I incisal relationship (Fig. 4). There was a thin periodontal phenotype and relatively high lip line. Tooth 21 had no swelling or sinus and was functional, with no mobility or aesthetic problems. It was not tender to percussion or buccal palpation. A ‘catch' was felt when probing the palatal aspect of 21; however, there was no pocketing, and the gingival cuff appeared healthy and tight. Pulp sensibility testing (Endo-Frost, Coltene, Germany) generated a positive response and a paralleling periapical radiograph (KaVo focus unit, Planmecca Romexis, USA) highlighted root end blunting and the appearance of resorption in the mid-third of tooth 21 (Fig. 5). Differential diagnoses included ECR or ITR. The extent of resorption was questioned because of the ‘catch' detected palatally. A CBCT (KaVo Vision V17, Planmecca Romexis, USA) of the anterior maxilla was therefore taken, revealing irregular enlargement of the root canal system in the cervical third, notably palatally, extending into the clinical crown and a radiolucent channel with clear exposure of the root canal system at the mid-third of the root canal (Fig. 6). A periapical radiograph taken at one year to evaluate progression of the lesion revealed no obvious changes. Correlation of findings led to a diagnosis of ITR of tooth 21 with a ‘normal pulp', in accordance with the American Association of Endodontists Diagnostic Terminology.^28^

**Fig. 7:**
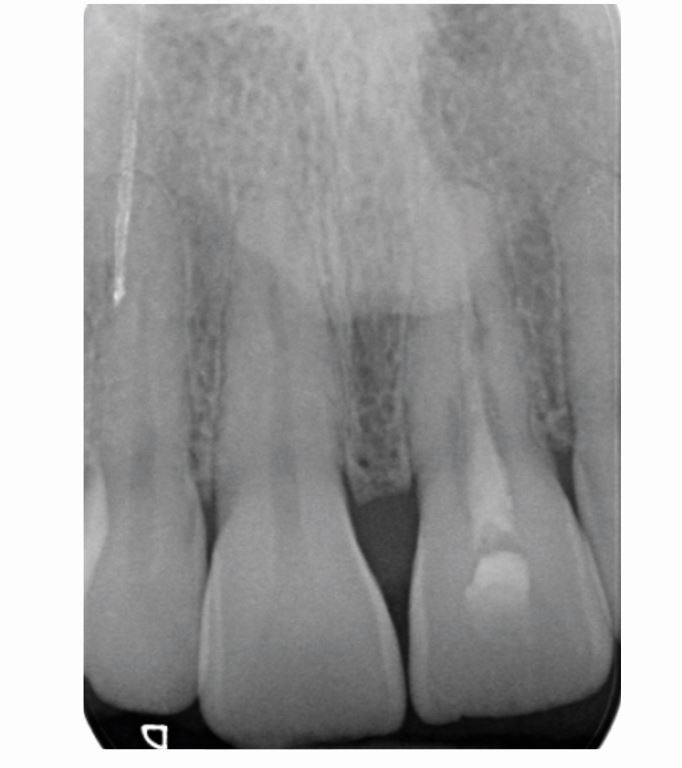
Intra-operative periapical radiograph of 21 taken to ensure calcium hydroxide is contained within the root canal confines. Irregular, tunnelling appearance of root canal system evident

**Fig. 8:**
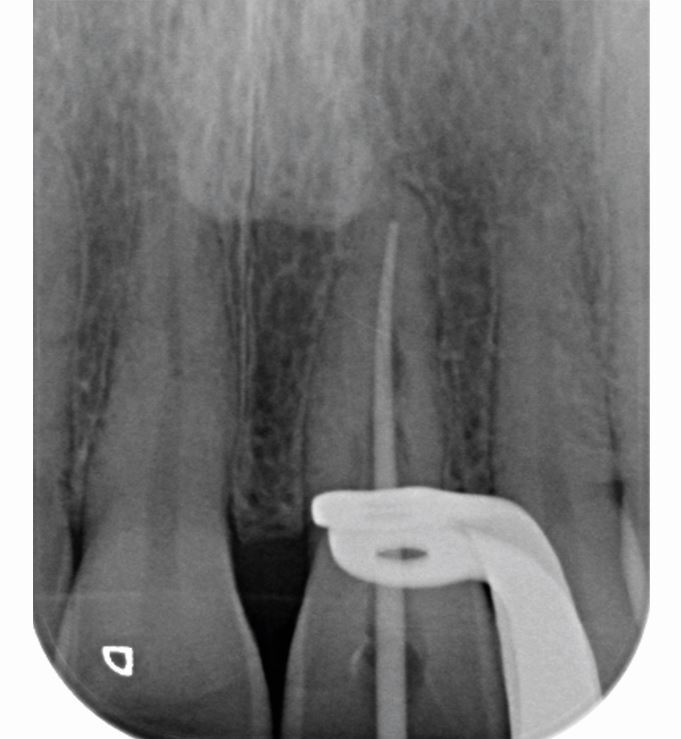
Master cone radiograph of tooth 21, taken 27 June 2022

**Fig. 9:**
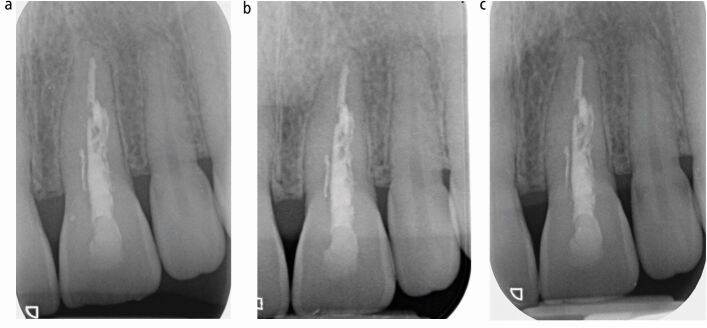
a) Immediate post-obturation radiograph of 21 (27 June 2022). b) Seven months postoperative radiograph of 21 (30 January 2023). c) 16 months postoperative radiograph of 21 (24 October 2023)

Treatment options (see online Supplementary Table 1) were discussed with the patient following which she consented for non-surgical endodontic treatment. Active treatment was chosen by the patient as an alternative to monitoring, as the tooth had become symptomatic and communication to the periodontium due to perforation mid root was suspected. Treatment was carried out using 2% lidocaine, 1:80,000 adrenaline, dental dam isolation (Coltene, Germany) and magnification (Dental Operating Microscope, Zeiss, Germany) over two appointments. At the first appointment, extirpation and root canal preparation using ProTaper Gold rotary files (Dentsply, Sirona, USA) and Calasept 3% sodium hypochlorite (Directa AB, Sweden) irrigation was carried out. The canal was dried using ProTaper Gold absorbent points (Dentsply Sirona, USA) and non-setting calcium hydroxide (Ultracal, Ultradent, USA) was injected as an intracanal dressing using a tip (NaviTips, Ultradent, USA). A periapical radiograph confirmed the calcium hydroxide was contained within the root canal confines (Fig. 7). Three weeks later, at the second appointment, the canal shaping was completed to a F3 ProTaper Gold (Dentsply Sirona, USA). Canal preparation allowed adequate irrigation within 1 mm of the working length, using a 27-gauge Luer Lock syringe, and a master cone radiograph was taken to confirm obturation was likely to be within 2 mm of the radiographic apex (Fig. 8). Final irrigation using 40% citric acid (Cerkamed, Poland), followed by Calasept 3% sodium hypochlorite (Directa AB, Sweden), was performed and the dried root canal obturated using a CSBS (TotalFill BC Sealer HiFlow, FKG Dentaire Switzwerland) and continuous wave compaction using F3 gutta percha cone and backfilled gutta percha. The endodontic access cavity was restored with Smart Dentine Replacement (Dentsply Sirona, USA). Figure 9a shows the immediate post-obturation radiograph. Radiographic review occurred at 7 and 16 months, postoperatively (Fig. 9b, Fig. 9c). No deleterious clinical signs or symptoms were noted, although the patient reported persisting low-grade symptoms (not affecting her quality of life) associated with 21 at seven months. A periapical radiograph taken to investigate the symptoms did not show any features warranting further intervention. Absence of symptoms and a further periapical radiograph at 16 months postoperatively confirmed no further indication for intervention. This case has resulted in the maintenance of tooth 21, which was the patient's priority.

## Discussion

In this case report, the patient had no known history of TDI and the aetiology of the ITR is unknown. However, publications highlight orthodontically induced inflammatory root resorption can occur in 19-31.4% of all patients undergoing fixed appliance orthodontic treatment.^29^ Most orthodontically induced root resorption affects the external root end surface because orthodontic forces compress the periodontal ligament (PDL), but intracanal changes cannot be excluded. However, in orthodontic-related resorption, the pulp is usually healthy and the resorption unrelated to the pulpal status but rather related to the tooth movement.

A possible subgingival cervical defect identified palatally on tooth 21 led to a provisional diagnosis of ECR before 3D imaging. The CBCT imaging was reviewed with a consultant radiologist. Due to perforation of the root canal at the mid-third level of the root and further expansion of the pulp chamber in the cervical third of the root without clear presence of pericanalar resorption resistant sheet (PRRS), the pattern of resorption was not in keeping with ECR. Furthermore, the clinical signs and symptoms that the patient presented with did not correlate with late stage ECR where the PRRS is lost. The enlargement of the pulp chamber in the cervical third of the root could have been in keeping with an internal inflammatory root resorption process; however, the clear radiolucent channel and communication with PDL space present in the mid-third of the root does not coincide the classic radiographic features of IRR. Despite a lack of clear aetiology from the patient's history and based on the clinical and radiographic findings, the diagnosis by exclusion was most in keeping with an internal tunnelling root resorption process. This presents with a disorganised pattern of resorption, as highlighted on the CBCT (Fig. 6). The potential for surgical investigation/repair was discussed with the patient; however, the abscence of periodontal involvement and no identified radiolucency at the site of PDL communication from this CBCT imaging questions the benefit of an invasive procedure. Reparative endodontic treatment of resorption defects is a novel technique discussed in case reports, whereby healthy pulp tissue apical of a resorption defect may induce dentine-like, hard tissue repair following the removal of infected tissue and disinfection. Resorption will only progress when microorganisms and their metabolites are not sufficiently removed; however, the requirements and optimum treatment protocol for reparative treatment remains to be clarified.^30^ There is conflicting evidence for the advantages of single appointment versus multiple appointments for completion of endodontic treatment.^31^ The decision to complete treatment over two visits was made due to the challenges disinfecting the complex anatomy caused by the ITR. A two-staged approach allowed the use of an interappointment intracanal medicament (non-setting calcium hydroxide) to enhance the disinfection process^32^ by accessing complex anatomical areas inadequately disinfected in a single visit with chemo-mechanical preparation.^33^An alternative to non-setting calcium hydroxide as an intracanal medicament in resorption cases is a corticosteroid and antibiotic-based preparation, such as Ledermix paste (Haupt Pharma GmbH, Wolfratshausen, Germany). In ITR cases, the use of Ledermix offers an anticlastic action and anti-inflammatory properties;^34^ however, its antimicrobial activity is limited and demeclocycline concentration reduces by tenfold after a week of application.^35^ Therefore, non-setting calcium hydroxide was preferred due to its long-term antimicrobial action. CSBSs have high levels of hydrophilicity and biocompatibility and set by reacting with water or under humid conditions. In this case, the use of a CSBS was preferable due to its fluidity and ability to promote an adequate seal if a perforation proved present to prevent subsequent inflammation and promote new cellular cemental apposition along with re-establishment of periodontal tissues.^26^ Furthermore, as the complex root canal anatomy resulting from ITR required sealing using a technique likely to fill the canal irregularities, a hybrid technique using continuous wave compaction with gutta percha and Totalfill BC highflow sealer (FKG, Dentaire, Switzerland) was chosen.^36^^,^^37^

The diagnosis of ‘normal pulp' in this case raises the issue of endodontic diagnostic terminology. A recent letter from Galicia *et al.*^38^ highlighted the need for diagnostic classification to represent the biological status of the dental pulp more accurately to determine its viability in a clinical context and to determine the appropriate treatment regime. Furthermore, an international survey about diagnostic terms used in endodontics highlighted a lack of consensus associated with the use of appropriate diagnostic terms for conditions. The authors concluded that further diagnostic terms and a modification of those in current use may be required regarding pulpal and periapical status.^39^

There is no specific classification of ITR known to the authors, which is attributed to the rare nature of ITR and the difficulties in diagnosis and establishing the extent of defects without imaging. A classification system, devised from clinical and radiographic findings, would facilitate effective and accurate communication of ITR lesions between colleagues and inform treatment planning regarding tooth prognosis, aiding the process of informed consent. Such classification is available for ECR. The Heithersay classification^40^ divides ECR lesions based on penetration of the lesion into coronal and root dentine using periapical radiographs. A CBCT-based classification for ECR which considers lesion height, circumferential spread and proximity to the root canal has also been developed, allowing the effect of the nature of ECR on the outcome of treatment to be assessed objectively.^41^ If ITR cannot be accurately classified, this limits the ability to assess the treatment outcome based on the extent of the lesion.

## Conclusion

This case discusses the published literature and management of a rarely seen clinical condition, ITR, which has a limited evidence-base guiding clinical management. Various forms of pathological resorption can occur; however, ITR differs from others in the multiple ‘tunnelling' defects created. This poses difficulties in accessing such anatomy with materials and equipment to sufficiently reduce and/or eliminate organic matter, biofilm and microorganisms to stop the resorptive process. In such cases, CBCT plays an important role in diagnosing the extent and exact location of the defect and whether treatment to restore the defect may be possible. Modern, bioactive and biocompatible materials are likely to impact the management of ITR cases and treatment outcomes; however, ITR cases are rare, rendering large-scale studies impossible, and evidence-based guidance on what to use, when to intervene and how best to do this, challenging to produce. It is therefore important that case reports, such as this one, are shared to inform clinicians on the management, materials and techniques that can be employed, including the use of CSBSs and thermal obturation, in efforts to ensure good-quality and lasting treatment outcomes in such cases. Going forward, a classification for ITR in addition to further diagnostic terminology in endodontics may be beneficial when managing such cases.

### Supplementary Information

Supplementary Table 1 (PDF 221KB)
