# Supplementary material for: Management of rarely seen internal tunnelling root resorption associated with a maxillary permanent incisor
Source: Br Dent J. 2024 Jun 28;236(12):955–61. doi: 10.1038/s41415-024-7504-7 (PMC11213701; doi:10.1038/s41415-024-7504-7)
Supplement: Supplementary file 1 — Supplementary Table 1 (PDF 221KB) [file 41415_2024_7504_MOESM1_ESM.pdf]

Table S1: Summary of IRR subtypes, clinical and radiographic presentation and treatment options

<table>
<tr><th>Internal Root Resorption types</th><th>Favourable</th><th>Questionable</th><th>Unfavourable</th></tr>
<tr><td rowspan="3">Surface:
Minor areas of resorption of dentinal walls in the root canal
Rare, transient and self-limiting
Aetiology: Unknown, likely minor trauma
Clinicians should be aware of this concept, as this condition can progress to Internal Inflammatory Resorption</td><td>Clinical presentation:
Asymptomatic
Undetectable clinically</td><td></td><td></td></tr>
<tr><td>Radiographic presentation:
Not evident due to the very minor and shallow defects</td><td></td><td></td></tr>
<tr><td>Treatment options:
No treatment required</td><td></td><td></td></tr>
</table>

Internal Root Resorption types	Favourable	Questionable	Unfavourable

<table>
<tr><td rowspan="3">Inflammatory:
Inflammatory process within a portion of the dental pulp which leads to loss of dentine from the pulp-facing root canal wall.
Active/ongoing, until the entire pulp necroses.
Aetiology: Trauma and/or caries</td>
<td>Clinical presentation:
Likely asymptomatic
If pulp necrosis, possible chronic apical abscess presentation (sinus tract)</td>
<td>Clinical presentation:
Possibly asymptomatic
If perforation / pulp necrosis, possible chronic apical abscess presentation (sinus tract)</td>
<td>Clinical presentation:
Possibly asymptomatic
If perforation / pulp necrosis, possible chronic apical abscess presentation (sinus tract)</td></tr>
<tr><td>Radiographic presentation:
An oval or circular shaped radiolucency (ballooning) of the root canal outline without any suggestion of perforation
CBCT may help with treatment planning and prognosis</td>
<td>Radiographic presentation:
An oval or circular shaped radiolucency (ballooning) of the root canal outline, may suggest perforation.
CBCT may help with treatment planning and prognosis</td>
<td>Radiographic presentation:
An oval or circular shaped radiolucency (ballooning) of the root canal outline with explicit perforation/extensive root destruction
CBCT may help with treatment planning and prognosis</td></tr>
<tr><td>Treatment options:
No active treatment and monitoring
If still active, root canal dressing with antibiotic-corticosteroid paste (for one week) / non setting calcium hydroxide (for up to 3 months)</td>
<td>Treatment options:
No active treatment and monitoring
Root Canal Treatment (3D obturation with heated technique and calcium-silicate based cement) with internal repair of perforation (if present)</td>
<td>Treatment options:
No active treatment and monitoring, accept likely loss of tooth
Extraction</td></tr>
</table>

	• Root Canal Treatment with heated obturation<br>• Extraction	• Root Canal Treatment (3D obturation with heated technique and calcium-silicate based cement) with surgical repair of perforation (if present)<br>• Extraction	

**Internal Root Resorption types**	**Favourable**	**Questionable**	**Unfavourable**

**Replacement**:<br>• Pulp and dentine tissues replaced with bone-like tissues in the root canal<br>• Very rare<br>• ***Aetiology***: Trauma/insult to the pulp	**Clinical presentation:**<br>• Most likely asymptomatic, discolouration or pink hue may appear if the resorption process reaches the coronal pulp region	**Clinical presentation:**<br>• Likely asymptomatic, discolouration or pink hue may appear if the resorption process reaches the coronal pulp region<br>• If perforation / pulp necrosis, possible chronic apical abscess presentation (sinus tract)	**Clinical presentation:**<br>• Likely asymptomatic, discolouration or pink hue may appear if the resorption process reaches the coronal pulp region<br>• If perforation / pulp necrosis, possible chronic apical abscess presentation (sinus tract)
	**Radiographic presentation:**<br>• Irregularly shaped radiolucency with mottled or cloudy appearance with a (partially) unclear outline.<br>• CBCT may help with treatment planning and prognosis	**Radiographic presentation:**<br>• Irregularly shaped radiolucency with mottled or cloudy appearance with a (partially) unclear outline.<br>• Root canal wall perforation may occur<br>• CBCT may help with treatment planning and prognosis	**Radiographic presentation:**<br>• Irregularly shaped radiolucency with mottled or cloudy appearance with a (partially) unclear outline.<br>• Evidence of root canal wall perforation<br>• CBCT may help with treatment planning and prognosis
	**Treatment options:**<br>• No active treatment and monitoring<br>• Root Canal Treatment (3D obturation with heated	**Treatment options:**<br>• No active treatment and monitoring<br>• Root Canal Treatment (3D obturation with heated technique and calcium-	**Treatment options:**<br>• No active treatment and monitoring, accept likely loss of tooth<br>• Extraction

	technique and calcium-silicate based cement)<br>• Extraction	silicate based cement) with internal repair of perforation.<br>• Root Canal Treatment (3D obturation with heated technique and calcium-silicate based cement) with surgical repair of perforation.<br>• Extraction	

**Internal Root Resorption types**	**Favourable**	**Questionable**	**Unfavourable**
**Replacement**:<br><br>• **Tunnelling:**<br><br>• Tunnelling resorption which burrows behind the predentine layer, adjacent to the root canal, with concomitant deposition of cancellous bone-like tissues<br>• Very rare variant of replacement resorption<br>• ***Aetiology:*** Trauma/Unknown, more likely associated with root fracture	**Clinical presentation:**<br><br>• Most likely asymptomatic, discolouration or pink hue may appear if the resorption process reaches the coronal pulp region	**Clinical presentation:**<br><br>• Likely asymptomatic, discolouration or pink hue may appear if the resorption process reaches the coronal pulp region<br>• If perforation / pulp necrosis, possible chronic apical abscess presentation (sinus tract)	**Clinical presentation:**<br><br>• Likely asymptomatic, discolouration or pink hue may appear if the resorption process reaches the coronal pulp region<br>• If perforation / pulp necrosis, possible chronic apical abscess presentation (sinus tract)
	**Radiographic presentation:**<br><br>• Limited resorption burrowing behind the root canal walls towards the cementum, no signs of perforation<br>• CBCT may help with treatment planning and prognosis	**Radiographic presentation:**<br><br>• Resorption burrowing behind the root canal walls towards the cementum with close proximity/perforation<br>• CBCT may help with treatment planning and prognosis	**Radiographic presentation:**<br><br>• Resorption burrowing behind the root canal walls towards the cementum with clear perforation<br>• CBCT may help with treatment planning and prognosis
	**Treatment options:**<br><br>• No active treatment and monitoring<br>• Root Canal Treatment (3D obturation with heated	**Treatment options:**<br><br>• No active treatment and monitoring<br>• Root Canal Treatment (3D obturation with heated technique and calcium-	**Treatment options:**<br><br>• No active treatment and monitoring, accept likely loss of tooth<br>• Extraction

	technique and calcium-silicate based cement)<br>• Extraction	silicate based cement) with internal repair of perforation.<br>• Root Canal Treatment (3D obturation with heated technique and calcium-silicate based cement) with surgical repair of perforation<br>• Extraction	
